# Effects of Functionalized Fullerenes on ROS Homeostasis Determine Their Cytoprotective or Cytotoxic Properties

**DOI:** 10.3390/nano10071405

**Published:** 2020-07-19

**Authors:** Svetlana V. Kostyuk, Elena V. Proskurnina, Ekaterina A. Savinova, Elizaveta S. Ershova, Olga A. Kraevaya, Larisa V. Kameneva, Pavel E. Umryukhin, Olga A. Dolgikh, Sergey I. Kutsev, Pavel A. Troshin, Natalia N. Veiko

**Affiliations:** 1 Laboratory of Molecular Biology, Research Centre for Medical Genetics, ul Moskvorechye 1, 115522 Moscow, Russia; svet-vk@yandex.ru (S.V.K.); savinova.ekaterina96@yandex.ru (E.A.S.); es-ershova@rambler.ru (E.S.E.); kamlar@med-gen.ru (L.V.K.); pavelum@mail.ru (P.E.U.); dolgiko@med-gen.ru (O.A.D.); kutsev@mail.ru (S.I.K.); satelit32006@yandex.ru (N.N.V.); 2 Laboratory of Molecular and Genetic Mechanisms of Adaptation and Stress, Department of Normal Physiology, I.M. Sechenov First Moscow State Medical University (Sechenov University), ul. Mohovaya 11-5, 125007 Moscow, Russia; 3 Center for Energy Science and Technology, Skolkovo Institute of Science and Technology, ul. Nobelya 3, 143026 Moscow, Russia; okraevaya@inbox.ru (O.A.K.); troshin2003@inbox.ru (P.A.T.); 4 Laboratory of Functional Materials for Electronics and Medicine, Institute of Problems of Chemical Physics of Russian Academy of Sciences, Semenov Prospect 1, Chernogolovka, 142432 Moscow, Russia

**Keywords:** functionalized fullerenes, ROS homeostasis, oxidative stress, NOX4, ROS-signaling, oxidative DNA damage, cell proliferation, apoptosis, cytoprotection, cytotoxicity

## Abstract

Background: Functionalized fullerenes (FF) can be considered regulators of intracellular reactive oxygen species (ROS) homeostasis; their direct oxidative damage—as well as regulation of oxidant enzymes and signaling pathways—should be considered. Methods: Uptake of two water-soluble functionalized C_70_ fullerenes with different types of aromatic addends (ethylphenylmalonate and thienylacetate) in human fetal lung fibroblasts, intracellular ROS visualization, superoxide scavenging potential, NOX4 expression, NRF2 expression, oxidative DNA damage, repair genes, cell proliferation and cell cycle were studied. Results & conclusion: The intracellular effects of ethylphenylmalonate C_70_ derivative (FF1) can be explained in terms of upregulated NOX4 activity. The intracellular effects of thienylacetate C_70_ derivative (FF2) can be probably resulted from its superoxide scavenging potential and inhibition of lipid peroxidation. FF1 can be considered a NOX4 upregulator and potential cytotoxicant and FF2, as a superoxide scavenger and a potential cytoprotector.

## 1. Introduction

Reactive oxygen species (ROS) such as superoxide anion radical and hydrogen peroxide play an important role in regulating biologic processes in living systems. Many pathologic conditions are associated with oxidative stress that manifests itself through lipid peroxidation, protein oxidative modification, DNA oxidative damage and redox imbalance. Reactive oxygen species are closely related to cell proliferation, growth, death, redox-signaling, immune function, inflammation, carcinogenesis, aging, degenerative processes [1]. ROS homeostasis is maintained with various sources of ROS and antiradical protective network. In cells, the dominant pool of ROS is produced by mitochondrial and microsomal respiratory chains and enzymes such as NADPH-oxidases, cyclooxygenases, lipoxygenases and peroxidases. Through glutathione network and thiol biochemistry, ROS homeostasis is linked with redox-signaling [2]. ROS-dependent and redox-dependent signaling pathways include PI3K/AKT/mTOR [3], MAPK [4], HIFs [5], NF–kB [6], p53 [7] and NRF2 [8]. In many cases, just the antioxidant or oxidant potential of biochemically active substances serves as a basis of their regulation or modulation effects.

Nowadays, biomedical properties of functionalized fullerenes (FF) are of great interest from their potentialities in diagnostics and therapy of diseases. Structural and electronic special features of fullerenes make it possible to perform various chemical transformations and obtain water-soluble derivatives with targeted biochemical activity [9]. For today, antiviral, anticancer, cytoprotective, antiapoptotic, and many other effects of functionalized fullerenes have been proven [10,11].

Due to their electronic structure, fullerenes have a unique physicochemical feature to act both as oxidants and antioxidants. The ability of fullerenes to either quench or generate cell-damaging ROS, altogether with their small size and a high surface area, could be exploited in biomedicine [12].

Antioxidant potential of fullerenes can be quantitatively described by the number of available unpaired electrons [13]. ROS scavenging fullerenes are promising for treatment of ROS related degenerative disorders [14]. Generally, the antioxidant potential of fullerenes has been widely studied [14,15,16,17,18,19,20]. Both pristine and functionalized fullerenes had a protective effect in ROS-dependent experimental models of cell damage, but the toxic responses also occurred [21,22,23,24].

On the other hand, the unique electronic π-system of fullerenes makes them potential photosensitizers upon the absorption of UV or visible light. In the presence of molecular oxygen, fullerenes can oxidize targets by either singlet oxygen or other ROS [25,26]. ROS-induced cytotoxicity upon UV irradiation and the ability to induce cell death makes the fullerenes potential anticancer and toxic agents [27,28]. However, functionalized fullerenes can induce oxidative stress without UV irradiation. As an example, the apoptotic HL-60 cell death induced by a pyrrolidinium fullerene derivative was suggested to be mediated by ROS generated by this derivative [29].

The state-of-the art paradigm of oxidative stress considers two aspects: primary oxidative damage of biomolecules by toxic ROS and upregulation of oxidant enzymes or ROS-dependent signaling pathways. As an example, fullerene C_60_ improves the MAPK expression level and stem cell survival, proliferation and cardiomyogenesis; modulates cardiomyogenic differentiation; improves the expression of cardiomyocyte-specific proteins; at the genetic level improves the expression of cardiomyocyte-specific proteins; and promotes the formation of gap junction among cells [30]. Fullerene C_60_(OH)_22_ regulated the malignantly differentiated bone marrow-derived mesenchymal stem cells via the Erk- and p38-MAPK and its downstream NF–kB signal pathway, but in normal stem cells, regulation occurred only through Erk- and p38-MAPK activation [31].

Water-soluble C_70_ fullerene derivatives with eight symmetrically attached solubilizing addends represent a highly promising class of compounds with pronounced antiviral and antioxidant activity. For example, the derivative of fullerene C_70_ with attached residues of 4-phenylbutanoic acid effectively inhibited human immunodeficiency virus (HIV-1 and HIV-2) and several strains of Influenza virus (A, B) [32]. Compound FF2 discussed below was shown to inhibit HIV-1 and HIV-2 in low micromolar concentrations [33]. Fullerene derivative with 2-(3-phenylpropyl)malonic acid-based addends homological to compound FF1 demonstrated prolonged antioxidant activity in vitro [15]. Additionally, a fullerene functionalized with eight residues of 3-phenylpropanoic acid was reported as an efficient tyrosine phosphatase inhibitor with IC_50_ values in the high nanomolar range [34].

Here, we have studied the effects of these two promising water-soluble functionalized fullerenes C_70_ with different types of aromatic addends (ethylphenylmalonate and thienylacetate) on ROS homeostasis in human fetal lung fibroblasts in relation to: (1) cell toxicity, (2) ROS-scavenging potential, (3) NOX4 regulation, (4) NRF2 pathway modulation, (5) oxidative DNA damage and reparation and (6) cell proliferation and cell cycle.

## 2. Materials and Methods

### 2.1. Synthesis and Characterization of Functionalized Fullerenes

**Figure 1:**
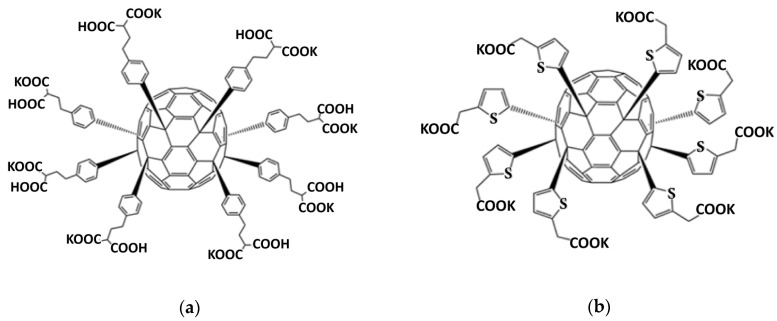
Molecular structures of (**a**) ethylphenylmalonate C_70_ derivative (FF1) and (**b**) thienylacetate C_70_ derivative (FF2) functionalized fullerenes (FF).

Compound FF1 (M = 2802 g/mol) (Figure 1a) was synthesized using Friedel–Crafts arylation reaction of C_70_Cl_8_ with dimethyl 2-phenethylmalonate in the presence of catalytic amount of FeCl_3_ followed by hydrolysis of ester groups as described previously [32]. Compound FF1, ethylphenylmalonate derivative, was characterized in the form of acid using NMR spectroscopy (^1^H, ^13^C). ^1^H NMR (500 MHz, (CD_3_)_2_SO, δ, ppm): 1.73–2.09 (m, 16H), 2.35–2.72 (m, 16H), 2.99–3.23 (m, 8H), 6.62–7.05 (m, 12H), 7.06–7.20 (m, 4H), 7.21–7.38 (m, 6H), 7.39–7.56 (m, 6H), 7.57–7.92 (m, 4H). ^13^C NMR (126 MHz, (CD_3_)_2_SO, δ, ppm): 30.84 (CH_2_), 32.49 (CH_2_), 33.18 (CH_2_), 51.16 (CH), 60.79 (C sp^3^ cage), 61.00 (C sp^3^ cage), 61.24 (C sp^3^ cage), 61.99 (C sp^3^ cage), 118.56, 127.85, 128.34, 128.68, 128.75, 128.87, 136.14, 136.45, 136.70, 136.97, 137.20, 140.60, 146.70, 147.07, 147.56, 147.78, 147.97, 148.44, 148.82, 149.04, 149.18, 149.25, 149.48, 149.95, 150.20, 150.31, 150.39, 151.07, 151.28, 151.37, 152.36, 152.66, 152.98, 153.30, 153.63, 153.90, 154.22, 154.33, 154.59, 154.66, 154.86, 155.29, 155.60, 156.37, 157.17, 158.16, 171.35 (COOH).

Compound FF2, thienylacetate derivative, (M = 2275 g/mol) (Figure 1b) was synthesized and characterized as reported previously [33]. Both compounds are highly soluble in water and the culture medium.

### 2.2. Cell Culture

Human fetal lung fibroblasts (HFLF) (the fourth cell passage) were provided by the Research Centre for Medical Genetics (RCMG). Approval#5 was obtained from the Committee for Medical and Health Research Ethics of RCMG. Cells were seeded at 1.7 × 10^4^ per mL in DMEM (Paneco, Moscow, Russia) with a 10% fetal calf serum (PAA Laboratories, Vienna, Austria), 50 U/mL penicillin, 50-μg/mL streptomycin and 10-μg/mL gentamycin (all the reagents were from Sigma-Aldrich, St. Louis, MO, USA) and cultured at 37 °C for 2 or 24 h as described elsewhere [35,36]. Fullerenes were dissolved under sterile conditions in a minimum volume of sterilized water or a saline solution. Before adding fullerene solutions to the cells, 5% ethyl alcohol (Sigma-Aldrich, St. Louis, MO, USA) was added. We have found previously that such an ethanol concentration does not affect the cells (data not shown). After adding the functionalized fullerenes to the medium, the cells were incubated for time intervals ranging from 15 min to 48 h.

### 2.3. MTT Assay

Cells were grown in a 96-well plate for 72 h. Cell viability was assessed with the 3-(4,5-dimethylthiazol-2-yl)-2,5-diphenyltetrazolium bromide (MTT) assay, as described previously [35,36]. The plates were read at 550 nm with EnSpire plate reader (EnSpire Equipment, Turku, Finland). MTT was purchased from Sigma-Aldrich, St. Louis, MO, USA.

### 2.4. Antibodies

Primary antibodies DyLight488-γH2AX (pSer139) (nb100-78356G NovusBio, Centennial, CO, USA), FITC-NRF2, (bs1074r-fitc, Bioss Antibodies, Inc. Woburn, MA, USA), FITC-BRCA1 (Nb100-598F, NovusBio, Centennial, CO, USA), PE-8-oxo-dG (sc-393871 PE, Santa Cruz Biotechnology, Dallas, TX, USA), CY5.5-NOX4 (bs-1091r-cy5-5, Bioss Antibodies, Inc. Woburn, MA, USA), A350-BCL2 (bs-15533r-a350, Bioss Antibodies, Inc. Woburn, MA, USA), BAX (Nb120-7977, NovusBio, Centennial, CO, USA) and secondary anti-rabbit IgG-FITC (sc-2359, Santa Cruz Biotechnology, Dallas, TX, USA) were used throughout.

### 2.5. Flow Cytometry Analysis (FCA)

Cells were washed with a Versene solution (Thermo Fisher Scientific, Waltham, MA, USA), treated with 0.25% trypsin (Paneco, Moscow, Russia), washed with the culture medium and suspended in phosphate buffer solution (pH 7.4) (Paneco, Moscow, Russia). The cells were fixed with paraformaldehyde (PFA, Sigma-Aldrich, Saint Louis, MO, USA) at 37 °C for 10 min, then washed three times with 0.5% BSA–PBS and permeabilized with 0.1% Triton X-100 (BSA and Triton X-100 were from Sigma-Aldrich, Saint Louis, MO, USA) in PBS for 15 min at 20 °C or with 90% methanol (Sigma-Aldrich, Saint Louis, MO, USA) at 4 °C, then washed with 0.5% BSA–PBS (3 times). The cells were stained with conjugated antibodies (1 μg/mL) for 2 h at room temperature, washed with PBS, and analyzed by a flow cytometer (CytoFlex S, Beckman Coulter, Brea, CA, USA). To stain with unconjugated primary BAX antibodies, the cells were incubated with antibodies (1 μg/mL) overnight (+4 °C), washed with 0.5% BSA–PBS, then incubated for 1 hour (at room temperature) with secondary antibodies (1 μg/mL), washed three times with 0.5% BSA–PBS, and analyzed.

For flow cytometry ROS analysis, unfixed cells suspension were incubated with 10-μM solution of H2DCFHDA in PBS (Molecular Probes/Invitrogen, Carlsbad, CA, USA) 15 min in the dark, washed PBS, resuspended in PBS and analyzed by flow cytometer in FITC channel.

### 2.6. Fluorescence Microscopy

An Axio Scope.A1 microscope (Carl Zeiss, Oberkochen, Germany) and a Leica TCS SP8 confocal microscopy platform (Leica Camera, Wetzlar, Germany) were used for fluorescent microscopy of stained cells.

### 2.7. Immunocytochemistry

Cells were grown in slide flasks (25 cm^3^, Thermo Fisher Scientific, Waltham, MA, USA), fixed in 3% paraformaldehyde at 4 °C for 20 min, washed with PBS and then permeabilized with 0.1% Triton X-100 in PBS for 15 min at room temperature, followed by blocking with 0.5% BSA in PBS for 1 h and incubation overnight at 4 °C with the antibodies. After washing with 0.1% Triton X-100 in PBS, fibroblasts were incubated for 2 h at room temperature with the FITC goat anti-mouse IgG, washed with PBS and then stained with DAPI (4′,6-diamidino-2-phenylindole) (Sigma-Aldrich, Saint Louis, MO, USA) as described in [35,36].

### 2.8. Reactive Oxygen Species Assays

An ROS analysis was performed with three techniques: flow cytometry, fluorescent microscopy and a total- fluorescence assay in a 96-well plate. Cells were grown in 96-well plates, incubated with the FFs, washed with PBS and treated with 10-μM solution of H2DCFHDA in PBS (Molecular Probes/Invitrogen, Carlsbad, CA, USA) and analyzed using the total fluorescence assay with a plate reader at λ_ex_ = 503 nm and λ_em_ = 524 nm (EnSpire Equipment, Turku, Finland) at 37 °C immediately and repeated every 5 min 10 times. The reaction rate constant for the formation of DCF (*k*) was calculated using the dependence of the DCF signal intensity on the time of cell incubation with H2DCFHDA. The data are presented as *k*_i_/*k*_0_ ratio, where *k*_i_ and *k*_0_ are the rate constants in the exposed and unexposed cells, respectively. The average value of the DCF signal for 16 wells ± standard deviation is reported.

### 2.9. NADH-Stimulated Lucigenin-Enhanced Chemiluminescence

The measurements were carried out with a 12-channel Lum-1200 chemiluminometer (DISoft, Moscow, Russia). Lucigenin (10,10-dimethyl-9,9-bacridinium dinitrate) and NADH were purchased from Sigma-Aldrich, Saint Louis, MO, USA. The cells were placed into a cuvette with a Krebs–Ringer buffer solution and lucigenin (0.4 mM). Chemiluminescence was recorded at 37 °C for 2 min, then NADH solution was added (0.8 mM) and the chemiluminescence was recorded for 15 min. From the chemiluminograms, the intensity of stimulated chemiluminescence *I*_NADH_ was calculated.

### 2.10. Quantification of mRNA Levels

Total mRNA was isolated using the RNeasy mini kit (Qiagen, Hilden, Germany), treated with DNAse I and then reverse transcribed by the reverse transcriptase kit (Sileks, Moscow, Russia). The expression profiles were obtained using qRT-PCR with SYBR Green PCR Master Mix (Applied Biosystems, Foster City, CA, USA). The mRNA levels were analyzed using StepOnePlus (Applied Biosystems, Foster City, CA, USA); the technical error was approximately 2%. TBP was used as a reference gene. The following primers were used (Sintol, Moscow, Russia): BRCA1 (F: TGTGAGGCACCTGTGGTGA, R: CAGCTCCTGGCACTGGTAGAG); NRF2 (NFE2 L2) (F: TCCAGTCAGAAACCAGTGGAT, R: GAATGTCTGCGCCAAA AGCTG); NOX4 (F: TTGGGGCTAGGATTGTGTCTA; R: GAGTGTTCGGCACATGGGTA); BRCA2 (F: CCTCTGCCCTTATCATCACTTT; R: CCAGATGATGTCTT CTCCATCC); CCND1 (F: TTCGTGGCCTCTAAGATGA AGG; R: GAGCAGCTCCATTTGCAGC); p21(CDKN1A) (F: TGTCCGTCAGAACCCATGC; R: AAAGTCGAAGTTCCATCGCTC); p16(CDKN2A) (F: ATGGAGCCTTCGGCTGACT; R: GTAACTATTCGGTGCGTTGGG); and TBP (reference gene) (F: GCCCGAAACGCCGAATAT, R: CCGTGGTTCGTGGCTCTCT).

### 2.11. Statistical Analysis

Experiments were repeated in triplicate. In FCA, the medians of the signal intensities were analyzed. Figures show the mean and standard deviation (SD). The significance of the observed differences was analyzed with nonparametric Mann–Whitney *U-*test. The *p*-values < 0.05 were considered statistically significant and marked on figures with “∗”. The data were analyzed with StatPlus2007 Professional 4.9.5 software (AnalystSoft Inc., Walnut, CA, USA, http://www.analystsoft.com).

## 3. Results

### 3.1. Fluorescence Spectra

**Figure 2:**
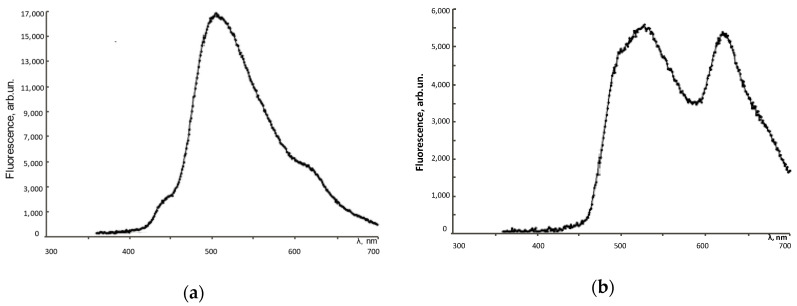
Fluorescence spectra for aqueous solutions of (**a**) FF1 and (**b**) FF2 at room temperature for the excitation at 370 nm. FF1 concentration is 178 nM; FF2 concentration is 220 nM.

The fluorescence spectra were recorded on CLARIOstar reader (BMG Labtech, Ortenberg, Germany). Both FF1 and FF2 exhibit intrinsic fluorescence in aqueous solutions and culture medium. Figure 2 shows the fluorescence spectra of FFs in an aqueous solution at room temperature with excitation at 370 nm. There are two fluorescence maxima at 505 and 620 nm for FF1 and 520 nm and 630 nm for FF2.

### 3.2. Cell Viability

**Figure 3:**
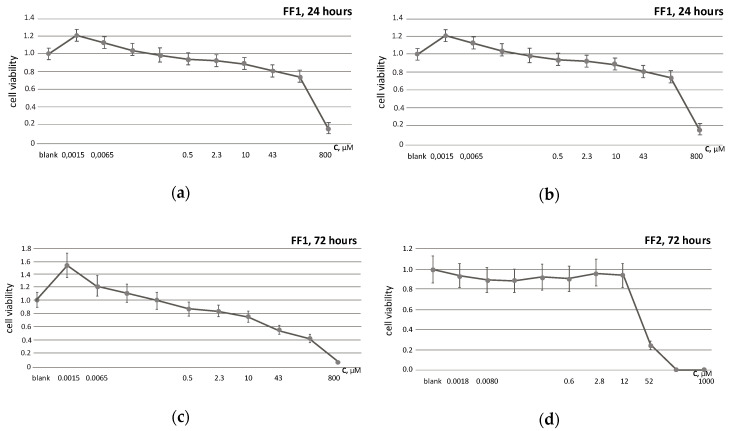
The 3-(4,5-dimethylthiazol-2-yl)-2,5-diphenyltetrazolium bromide (MTT) test: cell viability versus concentrations of FF1 in (**a**) 24 h and (**c**) 72 h; FF2 in (**b**) 24 h and (**d**) 72 h of incubation. In blank experiments, cells were incubated without the fullerenes.

Cell viability was examined by the conventional MTT assay. FF1 and FF2 effects were studied in concentrations 1.5 nM–0.8 mM and 1.8 nM–1.0 mM, respectively. The fullerenes were added to the medium at the beginning of the HFLF cultivation and incubated with the cells for 24 and 72 h. In 24 h, the cytotoxicity index IC_20_ (the concentration of the tested fullerene that is able to cause the death of 20% of the cells) for FF1 was 43 µM, for FF2, 13 µM. In 72 h, IC_20_ for FF1 was 2.3 µM, for FF2, 12 µM (Figure 3). Interestingly, FF1 became more toxic and FF2 became less toxic with time. Based on these data, we have selected the concentrations, where the toxic effect on the cells changed (the point of toxicity "bifurcation"). Thus, 6.5-nM FF1 was non-toxic both for 24 and 72 h of incubation, while 10-µM FF1 was non-toxic for 24 h and toxic for 72 h. Similarly, 8-nM FF2 was non-toxic both for 24 and 72 h of incubation, and 0.6-µM FF2 was toxic for 24 h and non-toxic for 72 h.

### 3.3. Uptake and Localization of FFs inside Human Fetal Lung Fibroblasts

**Figure 4:**
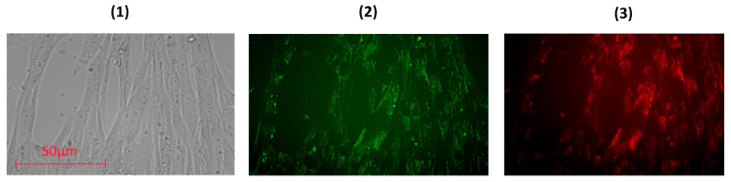
Fluorescence of FF1 (1 µM) in human fetal lung fibroblasts (HFLF) after 1 h of incubation. (**1**) Transmitted light; (**2**) fluorescence image detected with a 450–525-nm light filter; (**3**) fluorescence image detected with a 600–650-nm filter; excitation wavelength, 370 nm; magnification, ×40.

To study the penetration and distribution of the fullerenes in cells, confocal fluorescence imaging was used.-Light filters were selected in accordance with fluorescence spectra (Figure 2). The fluorescence experiments were performed in a 6-well plate at a cell concentration of 10^6^ cells/well. No less than 100 fields of view were analyzed; fluorescence intensity per a cell and the total fluorescence were analyzed using microscope software (ZEN 2 Core Imaging Software, Lux-Optic LH, Edegem, Belgium). As a result, FF1 permeated the cytoplasmic membrane quickly; after incubation within 1 h, its green and red intracellular fluorescence was detected (Figure 4).

**Figure 5:**
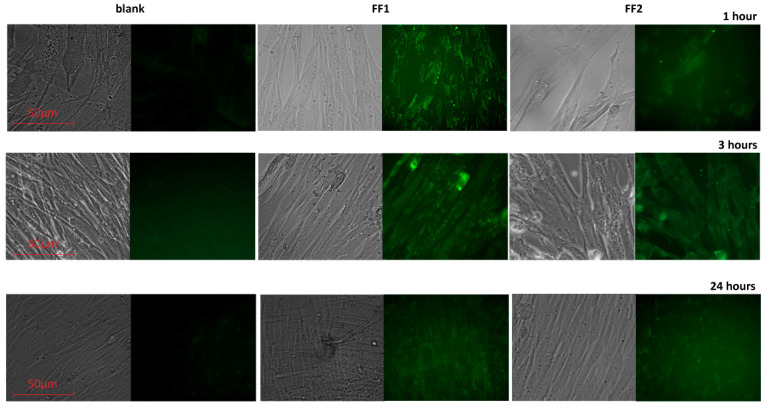
Fluorescence of FF1 and FF2 (1.3 µM) in cells for 1, 3 and 24 h of incubation: for each pair, the left image is transmitted light, the right image is the fluorescence of the compounds with a 450–525-nm filter; excitation wavelength 370 nm; magnification, ×40. In blank experiments, cells were incubated without the fullerenes.

The fluorescence of FF1 in HFLF remained high after 3 h of incubation and decreased gradually within 24 h (Figure 5).

**Figure 6:**
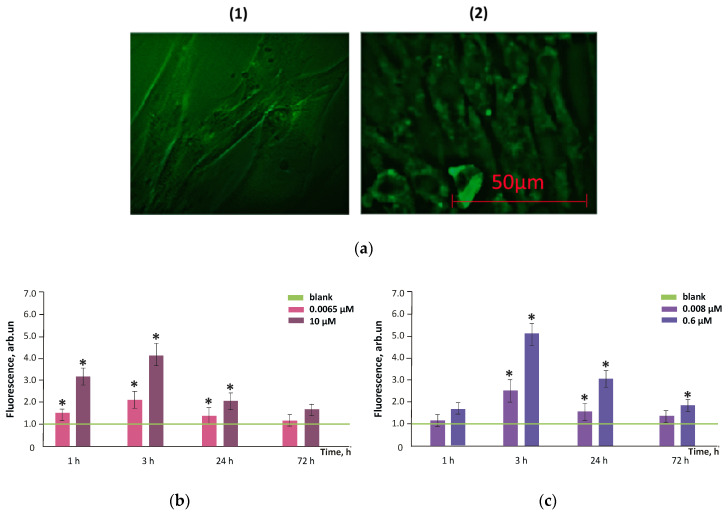
(**a**) FF2 fluorescence (1.3 µM) in cells during 1 (left) and 3 h (right) of incubation; a 450–525-nm filter; excitation wavelength, 370 nm, magnification, ×40; accumulation of FF1 (**b**) and FF2 (**c**) in the cells depending on incubation time determined with flow cytometry, concentrations are given in figure; (*) means that the results differ significantly from the blank (cells cultured without the fullerenes) according to the Mann–Whitney test (*p* < 0.05)

The FF2 distributed in the cell membrane after 1 h of incubation; it reached the cytoplasm of most HFLF cells after 3 h of incubation (Figure 6a). The FF2 fluorescence in cells decreased after 24 h (Figure 5) possibly indicating that the compound was leaving the cells or changing its structure.

The results obtained by fluorescence microscopy were confirmed by flow cytofluorimetry. The fluorescence of unfixed cells incubated with FF1 and FF2 for 1, 3, 24 and 72 h (the excitation wavelength, 370 nm) was proportional to the fullerene concentration (Figure 6b).

### 3.4. Intracellular ROS Visualization

To assess the amount of ROS in the cells, we used H2DCFHDA (dichlorodihydrofluorescein diacetate). This dye quickly permeates cell membranes and hydrolyzes to DCFH with hydrolases. Non-fluorescent DCFH is an intracellular probe sensitive to strong oxidants and H_2_O_2_ as they oxidize it to intensively fluorescing DCF.

**Figure 7:**
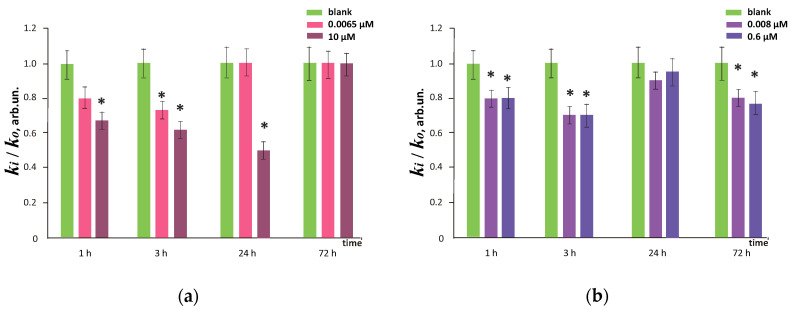
Reactive oxygen species (ROS) levels in cells expressed as a ratio of DCF synthesis rate constants after fullerene exposure—*k*_i_ and in blank—*k*_0_ at various (**a**) FF1 and (**b**) FF2 concentrations and time of incubation (indicated in figure). * means that the results differ significantly from the blank (the cells cultured without the fullerenes) according to the Mann–Whitney test (*p* < 0.05). In blank experiments, cells were incubated without the fullerenes.

Figure 7 shows the dependence of the ratio of DCF synthesis rate constants in cells for FF1 concentrations 6.5 nM and 10 µM and FF2 concentrations 8.0 nM and 0.6 µM to the blank values (the cells incubated without the fullerenes) for 1, 3, 24 and 72 h of incubation. The addition of 10-µM FF1 resulted in a statistically significant (35–40%) decrease in ROS level after 1, 3 and 24 h; the addition of 6.5-nM FF1 resulted in a statistically significant (≈20%) decrease in the ROS level after 1 and 3 h. After 72 h, the ROS level returned to the blank values (Figure 7a).

The FF2 in concentrations of 8.0 nM and 0.6 µM significantly reduced the ROS level (both by 20%) after 1, 3 and 72 h of incubation. After 24 h of incubation, a decrease in the ROS level was not significant relative to blank (Figure 7b).

### 3.5. Superoxide Scavenging Potential of FFs

**Figure 8:**
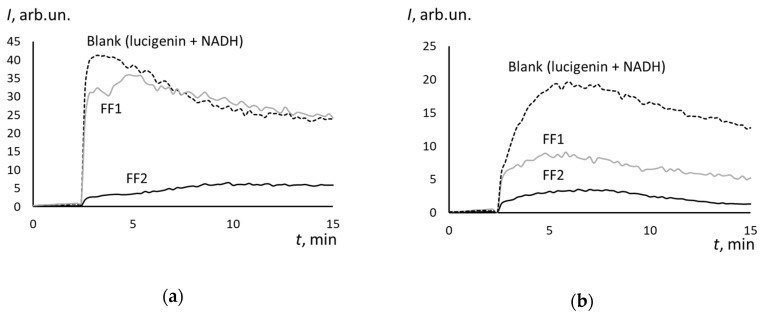
Effect of (**a**) FF1 (0.05 mM) and FF2 (0.30 mM) and (**b**) FF1 (0.30 mM) and FF2 (0.30 mM) on lucigenin-enhanced (0.40 mM) chemiluminescence in the presence of NADH (0.8 mM).

Figure 8 shows NADH-stimulated lucigenin-enhanced chemiluminescence kinetics in the presence of FF1 and FF2 of various concentrations. The chemiluminescence resulted from the reaction between lucigenin reduced by NADH–cytochrome b5 reductase and the oxygen with the formation of superoxide anion [37]. This system can be used for the superoxide scavenging ability of intracellular agents. Because of the low sensitivity of the method, we used 5-fold higher concentrations of fullerenes than in experiments with cells: 0.05-mM FF1 and 0.30-mM FF2 (Figure 8a). Moreover, we compared the antioxidant potential for equal concentrations of fullerenes (0.30 mM) (Figure 8b).

The results indicate that FF1 is a weak superoxide anion radical scavenger. Being taken in 6-fold deficiency compared to FF2, FF1 diminishes the blank chemiluminescence by 15%, while FF2 decreased the blank signal by 90%. In equal concentrations (0.30 mM), FF1 decreased the blank chemiluminescence by 55% compared to 90% by FF2. Thus, FF2 turns out to be a much more efficient scavenger of superoxide anion radical. In the future, it would be useful to study the antioxidant potential of FF2 in a lipid peroxidation system (for example, linoleic acid + Fe (II) or hemoglobin + coumarin 334) and TBARS method.

### 3.6. NOX4 Expression

The NADPH oxidases are main sources of ROS in cells. Today, NOX4 is of special interest as a ROS-homeostasis regulator. It needs no stimuli to work and produces hydrogen as a major product along with small amounts of superoxide anion radical. NOX4 protein was quantified with flow cytometry (Section 2.5 in Materials and methods). Gene expression was measured after 1, 3 and 24 h of incubation, while protein levels were measured after 1, 3, 24 and 72 h of incubation as protein levels increase often belatedly (they depend on both the efficiency of transcription and the rate of protein degradation). The protein levels confirm the changes in transcriptional activity of genes.

**Figure 9:**
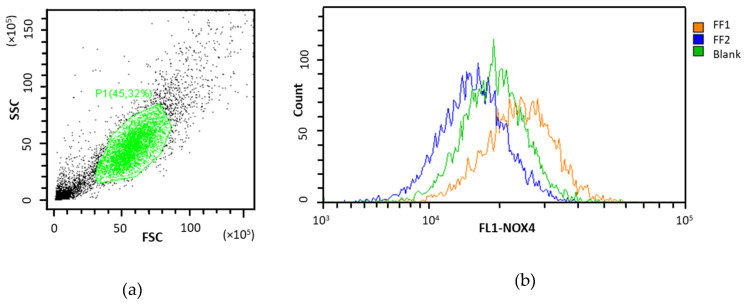
(**a**) Detection of human fetal lung fibroblasts according to FL1-NOX4 and (**b**) distribution of cells treated with FF1 and FF2 according to the FL1-NOX4. In blank experiments, cells were incubated without the FFs.

**Figure 10:**
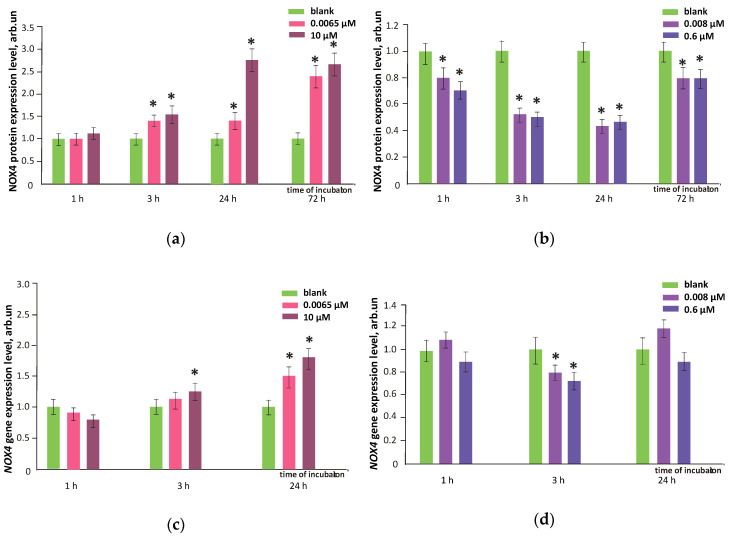

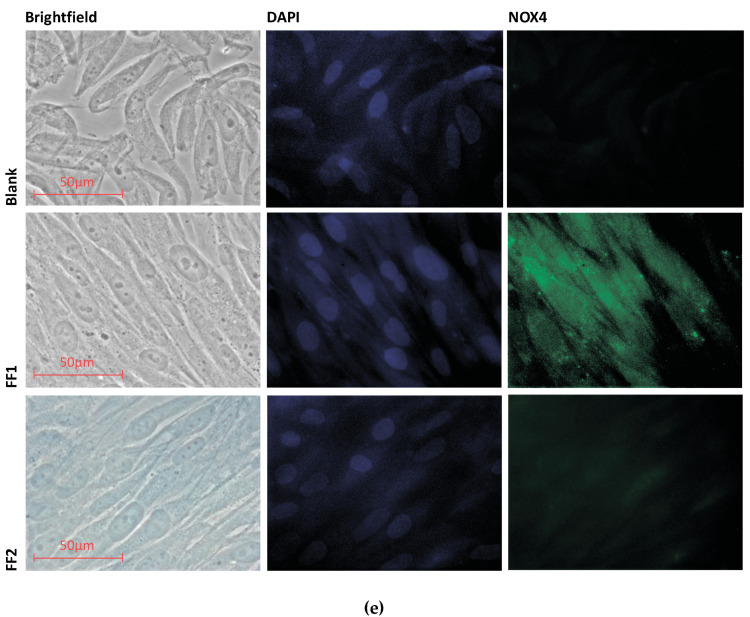
NOX4 protein level in HFLF treated with (**a**) FF1 and (**b**) FF2 in the concentrations shown in figure relative to the blank values (cells were cultured without fullerenes); the NOX4 gene expression in HFLF under (**c**) FF1 (**c**) and (**d**) FF2 in the concentrations shown in figure. NOX4 RNAs amount is the average of three experiments with fullerenes relative to the NOX4 gene expression in the blank. The TBP gene is used as the internal reference gene. (*) marks significant differences with blank cells, *p* < 0.01, nonparametric *U-*test; (**e**) fluorescence microscopy, fluorescence of NOX4 antibodies after addition of FF1 (10 µM) and FF2 (0.6 µM); from left to right: transmitted light, DAPI stained nuclei, and cells were treated with NOX antibodies; top to bottom: blank and cells after incubation with FF1 and FF2 for 24 h; magnification, ×20. In blank experiments, cells were incubated without the fullerenes.

We studied the effects of FF1 and FF2 in HFLF cells in relation to both gene NOX4 expression and NOX4 protein level. Raw data plots for NOX4 protein is presented as an example in Figure 9. After 24–72 h of incubation with FF1 (6.5 nM and 10 µM), NOX4 gene expression and protein level increased by 1.5–2.8 times, Figure 10 (1 and 2). An increase in the NOX4 protein expression in HFLF after 24 h incubation with FF1 may explain why the ROS level increased in 1–3 days. The delay of the NOX4 protein level relative to the gene expression after 1 h of incubation at a low FF2 concentration is most likely due to the complicated and multilevel regulation of gene expression. Here, a decrease in NOX4 protein level can result from decreased gene expression by 30–40% after 30–40 min of incubation (data are not shown).

Both NOX4 gene and protein expression in HFLF cells caused by FF2 in all studied concentrations remained low within 1–72 h. For nanomolar concentrations of FF2, a trend of increasing NOX4 gene expression was found (Figure 10a–d). The flow cytofluorimetry data for NOX4 protein were confirmed with fluorescence microscopy (Figure 10e).

Despite the antioxidant potential of FF1 and FF2, the gene and protein NOX4 expression increased within 24–72 h after adding FF1 to a HFLF culture. This leads to the intense production of intracellular ROS and a possible ‘late damage’ effect. FF2 did not influence NOX4 gene and protein expression after 72 h. In agreement with these results, H2DCFHDA experiments proved a prolonged antioxidant effect of FF2.

### 3.7. FFs Effects on NRF2 Pathway

**Figure 11:**
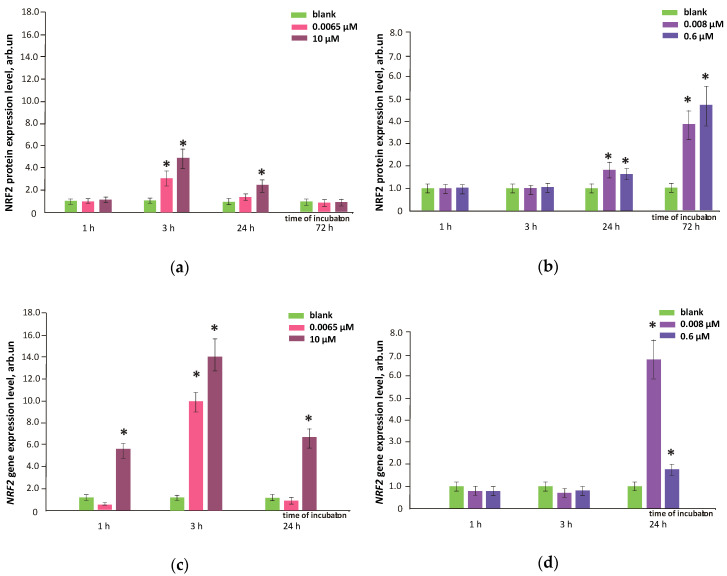

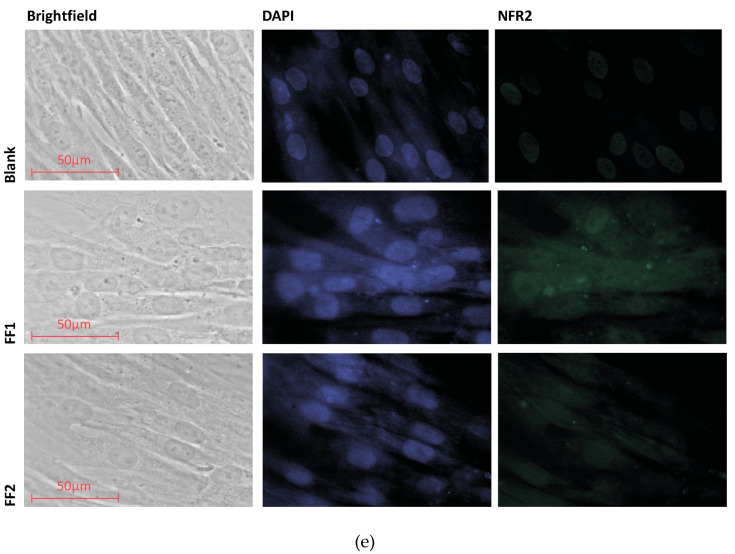
NRF2 protein level in HFLF treated with (**a**) FF1 and (**b**) FF2 in the concentrations shown in figure relative to the blank values (cells were cultured without fullerenes); NRF2 gene expression in HFLF after incubation with (**c**) FF1 and (**d**) FF2 in concentrations shown in figure. NRF2 RNA amount is the average of three experiments in the presence of fullerenes in relation to NRF2 expression in blank. TBP gene was used as the internal reference gene. (*) marks significant differences with blank (cells without fullerenes), *p* < 0.01, nonparametric *U-*test; (**e**) fluorescence microscopy, NRF2 antibodies fluorescence in cells treated with FF1 (10 µM) and FF2 (0.6 µM); from left to right: transmitted light, DAPI stained nuclei, and the cells were treated with NOX antibodies; from top to bottom: blank and cells after incubation with FF1 (10 µM) and FF2 (0.6 µM) for 24 h; magnification, ×40. In blank experiments, cells were incubated without the fullerenes.

NRF2 protein was quantified with flow cytometry (Section 2.5 in Materials and methods). Gene expression was measured with qRT-PCR (Section 2.10 in Materials and methods). The NRF2 transcription factor is involved in the cellular antioxidant response regulation. After 72 h, the NRF2 protein level activated by FF1 decreased and did not differ from the blank. the NRF2 protein level activated by FF2 increased by 3.5–4.5 times for both studied concentrations (Figure 11a, b). FF1 6.5 nM activated NRF2 gene expression by about 10 times after 3 h. FF1 10 µM activated NRF2 gene expression by 6–14 times after 1, 3 and 24 h (Figure 11c). NRF2 gene expression did not change in 1 and 3 h after adding FF2 to HFLF in the studied concentrations (Figure 11d). However, low NOX4 gene and protein levels during the first three hours of incubation should not promote ROS production, and there is no need in activating the antioxidant protection. After FF2 had been added to HFLF in 8.0 nM and 0.6 µM, NRF2 gene expression increased by 2–6.8 times within 24 h (Figure 11d).

The NRF2 protein expression data agree with the results obtained by flow cytofluorimetry and fluorescence microscopy. The NRF2 protein level increased by 5 times within 3 h after FF1 (10 µM) was added, by 2.5 and 1.8 times within 24 h after FF1 (10 µM) and the FF2 (0.6 µM) was added. The results were confirmed by the fluorescent microscopy (Figure 11e). In this case, NRF2 was localized both in the nucleus and in the cytoplasm.

Thus, the prolonged antioxidant FF2 effect on HFLF most likely results from a decrease in NOX4 expression in the early 1–3 h of the incubation and NRF2 transcription factor activation in the late hours of cultivation with FF2 (72 h). After adding FF1, an increased NRF2 expression probably decreased intracellular ROS within 1–24 h.

### 3.8. FFs Effects on Oxidative DNA Modifications, Double-Strand Breaks and Damage Response Genes Expression

**Figure 12:**
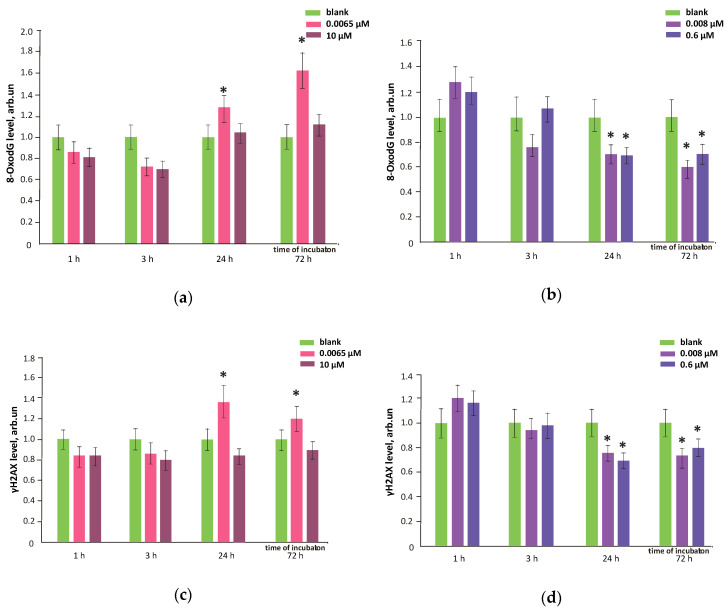
Mean values of 8-hydroxy-2’-deoxyguanosine, the marker of DNA oxidative damage and phosphorylated histone H2AX during incubation of HFLF with fullerenes (**a**,**c**) FF1 and (**b**,**d**) FF2 (concentrations are shown in figure). (*) marks the data significantly different from the blank (no fullerenes were added to the cells) according to the Mann–Whitney test. In blank experiments, cells were incubated without the fullerenes.

A marker of oxidative DNA damage, 8-oxo-dG, H2AX and BRCA protein were quantified with flow cytometry (Section 2.5 in Materials and methods). Gene expression was measured with qRT-PCR (Section 2.10 in Materials and methods). When FF1 (6.5 nM) was added to the cells, oxidative DNA modifications increased by 20–60% compared to the blank within 24–72 h (Figure 12a). FF1 10 µM did not cause oxidative DNA damage (Figure 12a), due to efficient ROS binding for 24 h.

A minor increase in the DNA oxidative modifications during the first hour after adding FF2 was probably due to a prolonged permeation through the cytoplasmic membrane and neutralization of lipid ROS. FF2 (8.0 nM and 0.6 µM) reduced the oxidative DNA damage by 20–40% within 24 and 72 hours after being added to the cells (Figure 12b). Thus, this substance may be considered as an efficient antioxidant with a prolonged action.

An increased amount of 8-hydroxy-2’-deoxyguanosine in the cell may cause the DNA breaks. One of the methods to assess double-stranded DNA breaks is based on the H2AX conservative histone protein involved in DNA chromatin packing. H2AX is phosphorylated in the serine 139 residue at the DNA break site involving ATM, ATR and DNA-PK kinases. The phosphorylated γH2AX histones bound with labeled antibodies are visualized in the cell nucleus. High levels of phosphorylated γH2AX histones indicates that the double-stranded DNA breaks in the cell nuclei.

**Figure 13:**
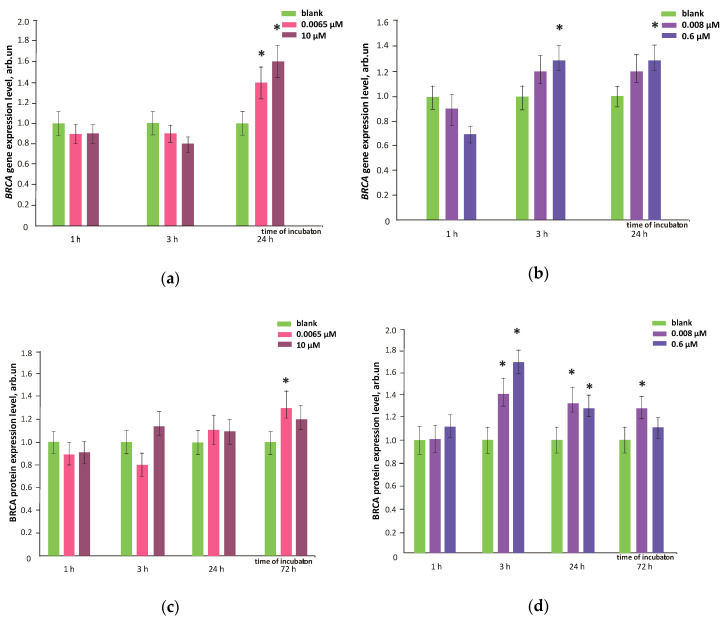
BRCA1 gene expression in HFLF cells caused by (**a**) FF1 and (**b**) FF2; BRCA1 protein levels in HFLF treated with (**c**) FF1 and (**d**) FF2 relative to blank (concentrations are shown in the figure). The mean of BRCA1 RNA amount of three experiments in relation to blank (BRCA1 gene expression in the cells with no fullerenes added). TBP gene was used as an internal reference gene. (*) marks significant differences in comparison with the blank, *p* < 0.01, nonparametric *U-*test. In blank experiments, cells were incubated without the fullerenes.

Proved with flow cytometry, FF1 (6.5 nM) increased the amount of double-stranded DNA breaks by 20–40% compared to the blank within 24–72 h (Figure 12c); 10 µM of FF1 did not influence the amount of DNA breaks (Figure 13c), which also correlates with the level of oxidative DNA damage. FF2 (8.0 nM and 0.6 µM) not only diminished the oxidative DNA damage within 24 and 72 h, but also reduced the amount of DNA double-strand breaks (Figure 12d). This fact is an evidence that FF2 may have the protective anti-genotoxic effect.

As a reaction to DNA damage, the DNA repairing genes can be activated such as BRCA1. FF1 (6.5 nM and 10 µM) increased BRCA1 expression by 20–40% within 24 h (Figure 13a). The BRCA1 protein level increased also within 72 h (Figure 13c). Probably, the expression of repair genes and proteins neutralizes the negative long-term (72 h) effect of this compound. FF2 (8.0 nM and 0.6 µM) not only diminished the oxidative damage and DNA breaks, but also increased by 30–60% BRCA1 gene expression within 3–72 h and the BRCA1 protein level within 24–72 h (Figure 13b,d) that resulted in a low level of double-strand DNA breaks.

### 3.9. FFs Effects on Cell Proliferation and Cell Cycle

DNA breaks can be repaired under the cell cycle stoppage. In addition, the cells may activate a programmed cell death (apoptosis). According to the MTT test results, we have not found the increase in proliferative activity of HFLF after incubation with FF2 (8.0 nM and 0.6 µM) within 3 days whereas FF1 (6.5 nM) increased the number of the cells by ≈ 20%, FF1 (10 μM) reduced the number of the cells population by ≈ 20% (Figure 3).

**Figure 14:**
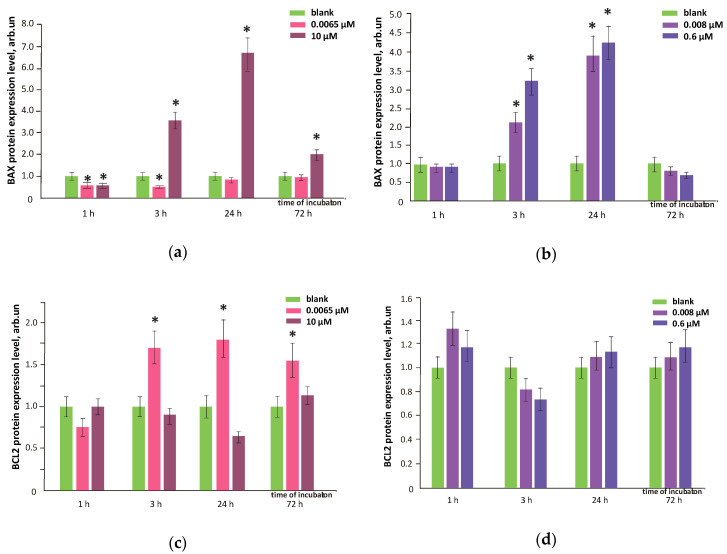
BAX and BCL2 proteins levels in HFLF culture treated with (**a**,**c**) FF1 and (**b**,**d**) FF2 (concentrations are shown in figure) relative to blank (cells with no fullerenes added). (*)— significant differences relate to blank, *p* < 0.01, nonparametric *U-*test. In blank experiments, cells were incubated without the fullerenes.

Apoptosis regulation is one of the key processes determining the number of cells. We studied the effects of FF1 and FF2 on the expression of the proapoptotic (BAX) and antiapoptotic (BCL2) proteins. All proteins were quantified with flow cytometry, the gene expression was measured with qRT-PCR. When added, FF1 (6.5 nM) increased the expression of antiapoptotic protein BCL2 by ≈1.5–1.8 times within 3–72 h, the proapoptotic protein BAX expression was inhibited or unchanged (Figure 14a,c). This demonstrates that apoptosis was inhibited by 6.5-nM FF1. The increasing number of HFLF exposed with nanomolar FF1 concentrations may be due to the inhibition of apoptosis at the level of gene regulation.

Treatment with FF1 (10 µM) resulted in a 2-fold increase in the proapoptotic BAX protein within 72 h (Figure 14a), which can decrease the number of cells by about 20% within 72 h (Figure 3).

A similar, but less marked trend was found in the case of incubation of the cells with FF2 (8.0 nM and 0.6 µM) within 72 h: the expression of the antiapoptotic protein BCL2 increased and proapoptotic protein BAX decreased (Figure 14c,d). Thus, the apoptosis was inhibited. A decrease in the expression of BAX protein because of incubation may be due to the increased expression of NRF2 gene [38].

The number of cells depends on the apoptosis and cell cycle progression. Here, we have studied the effects of FF1 and FF2 on the HFLF cell cycle. When the cell cycle is initiated, cyclin D1 protein synthesis encoded by the CCND1 gene is activated. Cyclin D1 triggers the G1 cell cycle phase and plays a key role in regulating the transition from G1 to S phase of the cycle. Cyclin-dependent kinase inhibitors are regulators of the cell cycle. The P21 kinase inhibitor has a negative effect on the cyclin-dependent kinase (CDK) complexes activity in G1 cell cycle phase; the P21 gene (CDKN1A gene) expression may lead to G1 arrest. The CDKN2A gene encodes the P16 protein, a cell cycle regulator that binds with CDK4 and CDK6 and disrupts cyclin D1 to CDK binding. The complex system of cell cycle gene regulation leads either to its progression or to its inhibition.

**Figure 15:**
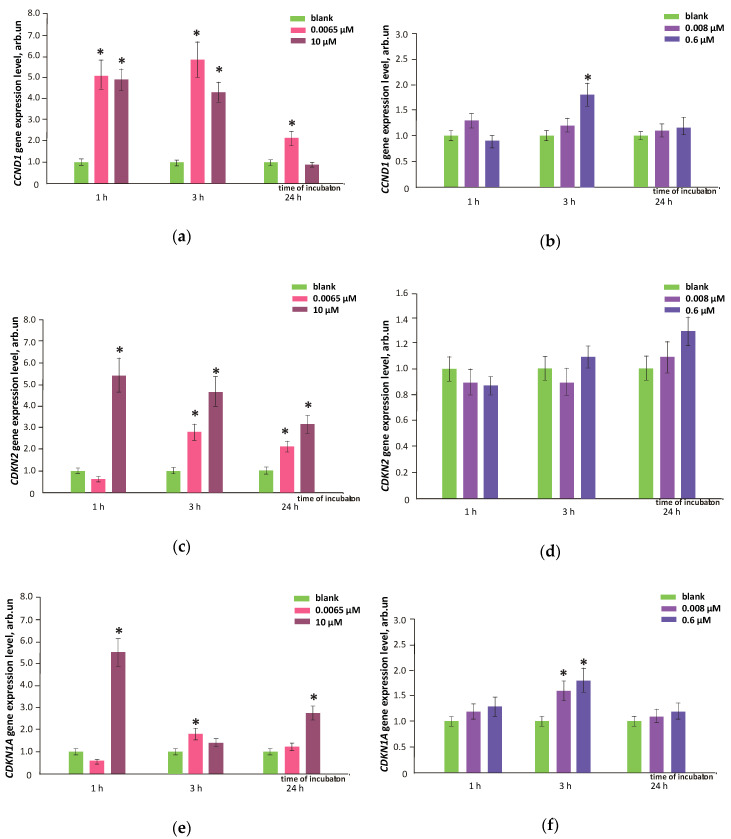

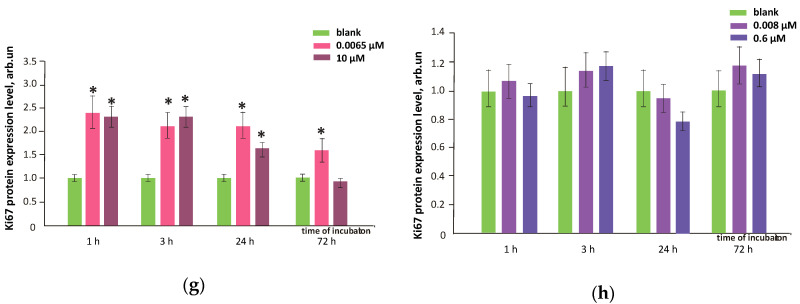
The expression of CCND1, CDKN2A and CDKN1A genes in HFLF as a result of treatment with (**a**,**c**,**e**) FF1 and (**b**,**d**,**f**) FF2 (concentrations are shown in figure). The RNA amount is the mean value of three experiments relative to blank. The TBP gene was used as an internal reference standard gene. Ki-67 protein level in HFLF treated with (**g**) FF1 and (**h**) FF2 (concentrations are shown in figure) relative to blank (cells with no fullerenes added). (*) marks significant differences with blank, *p* < 0.01, nonparametric *U-*test. In blank experiments, cells were incubated without the fullerenes.

The FF1 (6.5 nM) increased the expression of the CCND1 gene by 2–6 times within 24 h, while the expression of the CDKN1A and CDKN2 genes increased within 3–24 h (Figure 15a,c,e). As a result of the complicated interaction of genes that regulate the cell cycle, the cell cycle progression intensified. The increased HFLF proliferative activity was also manifested itself as an increase in Ki-67 level by 1.5–2.4 times within 1–72 h after the compound was added to the cells (Figure 15g).

FF1 (10 µM) led to an increase in the CCND1 gene RNA level by 4–5 times within 1–3 h; the expression of the CCND1 gene was inhibited within 24 h. The expression of the CDKN1A and CDKN2 genes increased by 3–6 times within 1–24 h (Figure 15a,c,e). The enhanced expression of the kinase inhibitors led to a decreased cell cycle progression. It also could explain a decrease in the number of HFLF by ≈ 20% within 3 days. The expression of Ki-67 proliferation marker increased by 1.5–2.5 times within 1–24 h and decreased only after 72 h (Figure 15 g).

FF2 (8.0 nM and 10.6 µM) caused no effect on the expression of the CCND1, CDKN1A and CDKN2 genes and on the concentration of Ki-67 proliferation marker (Figure 15b,d,f,h).

## 4. Discussion

Using the fluorescence of FF1 and FF2, we have studied their permeation into cells, accumulation and localization in the cytoplasm. FF1 quickly permeated the cytoplasmic membrane. Its intracellular fluorescence was detected within one hours. After one hours of incubation, FF2 was distributed in the cell membrane. It was found in the cytoplasm of most cells within three hours.

As for intracellular superoxide anion radical scavenging, FF2 had a more pronounced antioxidant potential compared to FF1. Taking into account its affinity to lipid membrane, we could consider it a potential lipid peroxidation inhibitor. However, according to the H2DCFHDA method, FF1 was found to be a more efficient H_2_O_2_ antioxidant. It is known that fluorescein is oxidized by strong oxidants such as peroxynitrite [39], as well as by intracellular hydrogen peroxide [40,41]. Oxidation is significantly enhanced by peroxidases, which oxidize fluorescein [42]. Superoxide anion radical, hydroxyl radical [43] and singlet oxygen [44] do not oxidize fluorescein. Even considering some difficulties in interpretation of results [45], we can state that FF1 is a more efficient antioxidant in relation of hydrogen peroxide that may be a factor of redox-signaling control [46,47].

After 24–72 h of incubation with FF1, an increased expression of the NOX4 gene and protein, which led to intracellular ROS production and to the development of a ‘late damage’ effect with an increased level of DNA oxidative modifications and double-strand breaks. The oxidative stress is likely to result in an increased NRF2 gene expression and decreased intracellular ROS within 1–24 h.

The FF2 did not increase the expression of NOX4 gene and protein within 72 h. Moreover, the NOX4 expression was even reduced within 1–3 h. Thus, FF2 demonstrated an inhibitory effect on NOX4. Together with its antioxidant potential, this phenomenon may explain the prolonged antioxidant effect. FF2 did not affect the NRF2 activation within early hours of incubation. NRF2 activation was found later (in 72 h). FF2 diminished oxidative DNA damage within 24 and 72 h after adding to the cells and reduced the double-strand breaks. All this allow us to consider effects of FF2 as genoprotective.

Based on MTT assay results, we did not find enhancing the HFLF proliferative activity within 3 days after adding FF2 (8.0 nM and 0.6 µM). FF1 (6.5 nM) increased the number of cells by ≈ 20% due to the activation of cell proliferation and inhibition of apoptosis. FF1 (10 µM) diminished the number of cells by ≈ 20% due to enhanced apoptosis and reduced HFLF proliferation.

**Figure 16:**
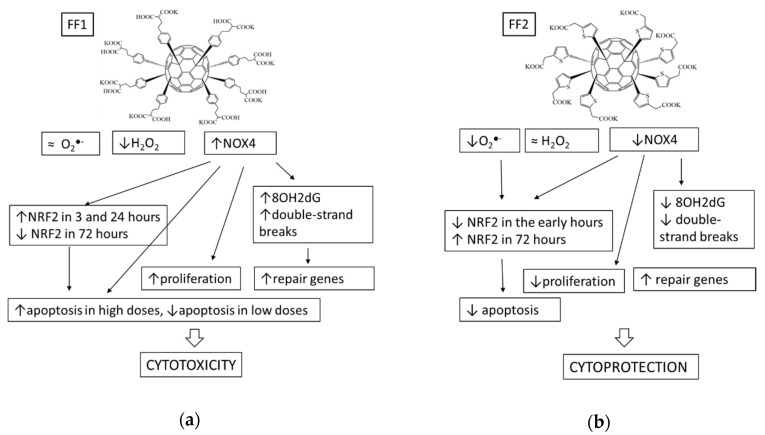
Schematic representation of mechanisms of cytotoxic or cytoprotective effects of (**a**) FF1 and (**b**) FF2.

Thus, both compounds were antioxidants in relation to ROS. FF1 had more pronounced effect on hydrogen peroxide, whereas FF2 was a scavenger of superoxide anion–radical and probably an inhibitor of lipid peroxidation. FF1 was significantly more toxic: it increased NOX4 expression, activated NRF2 pathway, enhanced oxidative DNA damage and double-strand breaks, activated repair gene expression, increased cell proliferation and affected complicatedly on apoptosis. These effects may be referred as oxidative stress, which can be caused by increased expression of NOX4. On the contrary, FF2 was less toxic. It practically did not affect the NOX4 activity, diminished oxidative DNA damage, double-strand breaks and repair gene activity, inhibited proliferation and apoptosis. Presumably, the key mechanism of the FF1 effect lies in the activation of NOX4, and the cytoprotective effects of FF2 were due to its antioxidant potential. The summarizing scheme was given in Figure 16.

The unique structure of NOX4 results in the constitutive generation of hydrogen peroxide as a main product [48,49]. NOX4 is the major source of oxidative stress in neurodegeneration on ischemic stroke [50], diabetes [51], cardiovascular diseases, pulmonary fibrosis and hypertension [52]. This enzyme is involved in oxygen sensing, vasomotor control, cellular proliferation, differentiation, migration, apoptosis, senescence, fibrosis, inflammation and angiogenesis [49]. The subcellular localization of NOX4 is cell type specific from mitochondria [53] to endoplasmic reticulum, nucleus and focal adhesions [49].

The regulation of NOX4 is a complex process which is far from being completely understood. Transcription of NOX4 is induced by chronic hypoxia, which was demonstrated in a mouse lung model; the transcription factors E2F1, HIF-1α and SMAD binding elements as well as signaling via NFκB and TGF-β/JNK have also been shown to influence NOX4 expression [54]. MicroRNAs can also regulate NOX4 expression by targeting the 3’-UTR, including MIR21A-3P, MIR25, MIR99A, MIR31 and MIR17 [55]. NOX4 is activated by cytokines such as TNF-α and IFN-γ [56]. NOX4 in colon cells was activated by ROS generation and subsequently impairs DNA to induce tumorigenesis via the insulin mediated PI3K/AKT pathway [57]. Interestingly that some quinone derivatives could modulate the NOX4 activity in different cellular models. NOX4 activity was also stimulated by reducing agents that possibly act by reducing the disulfide bridge (Cys226, Cys270) located in the extracellular E-loop of NOX4 [58]. The complexity and unexplored character of NOX4 regulation do not allow unambiguous conclusions about the FF1effects mechanisms; it is possible that a certain role may have a violation of the H_2_O_2_ and redox equilibrium balance.

The NOX4 can activate the Nrf2-regulated pathway in cardiomyocytes [59]. NOX4-derived hydrogen peroxide enhanced Nrf2 stability via disruption of redox-dependent proteasomal degradation and stimulated its activity through activation of PI3K-signaling; inhibition of Nrf2 could suppress cell growth and efficiently reverse the enhancement effect of NOX4 on cell growth [60]. Our data are consistent with the literature: in FF1case, the Nrf2 expression was increased, while FF2 had almost no effect on the NRF2 in early hours. However, the NRF2 activity increase is apparently unable to compensate oxidative stress and prevent the oxidative DNA damage. It is known that in contrast to the other NOXs, the NADPH oxidase NOX4 exists in the immediate environment of the nucleus and is involved in genomic instability as well as in cancer and other inflammation-related diseases [61,62]. This is how FF1genotoxic effect is realized.

Increased cell proliferation and NOX4 activation under FF1 is also consistent with the accepted concept that NADPH oxidase-derived reactive oxygen species are important for various cellular functions—including proliferation—though the mechanisms and targets of ROS signals are not well understood. It has been shown that NOX4 expression regulated by the noncanonical pathway ERK1/2 may be involved in TGF-beta1-induced proliferation of endothelial cells, which is vital during angiogenesis and vascular development [63]. Under normoxic and hypoxic conditions, NOX4 enhanced proliferation and inhibited apoptosis [64]. In vitro assays confirmed that knockdown of NOX4 expression blocked cell proliferation and the expression of Cyclin D1 and BAX [65]. In adventitial fibroblasts, NOX4 overexpression stimulated migration and proliferation, as well as matrix gene expression [66]. The authors suggested that H_2_O_2_-producing NADPH oxidases NOX4 and DUOX2 regulated cell cycle entry as part of a p53-dependent checkpoint for proliferation [67].

FF2 causes a decrease in NOX4 activity, oxidative DNA damage and the double-strand breaks number and inhibits cell proliferation and apoptosis. In the case of FF2, the inhibition of cell proliferation may be caused by a decreased NOX4 activity as well as the scavenging potential of this compound. Thus, FF2 can be considered as a promising antigenotoxic agent as other antioxidant drugs [68,69] or even inorganic nanoparticles [70].

Thus, the antioxidant potential, which depends on the chemical structure of fullerene, is one of the factors determining their cytoprotective properties. Importantly, the cell response can be realized by different mechanisms with different dynamics. Thus, the key factor of the prolonged cytoprotective action of FF2 is a low level of NOX4 and a late increase in NRF2, although the mechanism of such activation of NRF2 is still unclear. The cytotoxicity of FF1 results from an increase in NOX4 followed by the oxidative DNA modification and breaks, as well as the absence of late NRF2 activation. Obviously, FF2 is a much more promising cytoprotective agent; however, at nanomolar concentrations, FF1 can also be considered a short-acting antioxidant and cytoprotector.

## 5. Conclusions

The examined compounds can be considered regulators of intracellular ROS homeostasis, however, all intracellular effects of FF1 such as an activation of the NRF2 pathway, DNA damage and repair, cell proliferation and apoptosis can be explained in terms of upregulated NOX4 activity. The intracellular effects of FF2 can most probably result from its superoxide scavenging potential and possible inhibition of lipid peroxidation in membranes. To sum up, the FF1 compound can be considered a NOX4 upregulator and potential cytotoxicant and the FF2 compound, a superoxide scavenger and a potential cytoprotector.
